# Efficacy of an Abdominal Surgery Simulator in Didactic Medical Training: A Randomized Controlled Trial

**DOI:** 10.7759/cureus.77935

**Published:** 2025-01-24

**Authors:** Shea E Fincher, Victoria M Koniuk, Michael S Benavidez, Madison C Benefield, Danielle L Drew, Dean M Hasan, Nicholas Minner, Tara C Prakash, Michael J Parks, Tom Lindsey

**Affiliations:** 1 Simulation and Technology, Edward Via College of Osteopathic Medicine, Spartanburg, USA; 2 Surgery, Edward Via College of Osteopathic Medicine, Spartanburg, USA

**Keywords:** gastrointestinal surgical anatomy, medical education, medical training, simulation, surgical simulator

## Abstract

Introduction

Many medical students do not have access to hands-on surgical experience throughout the pre-clinical curriculum. To address this issue, we developed a low-cost abdominal surgery simulator for use during instructive years and tested its efficacy through a proof-of-concept, randomized controlled trial. Our goal is to help medical students integrate foundational anatomy with surgical pathology, enhancing their understanding and translating to academic success on board-relevant topics.

Methods

Second-year students at Edward Via College of Osteopathic Medicine-Carolinas Campus (VCOM-CC) were split into two groups. The control group utilized the traditional curriculum, while the experimental group used the curriculum along with the simulator in instructive and integrative sessions. We created pre- and post-assessments comprised of gastrointestinal anatomy relevant to United States Medical Licensing Examination (USMLE) Step 1 and Comprehensive Osteopathic Medical Licensing Examination (COMLEX) Level 1 medical licensing examinations and compared the groups’ outcomes using statistical analysis.

Results

Statistical analysis was performed on the average change between the pre-assessment and post-assessment scores. This trial revealed an average change of -0.267, a standard deviation of 3.90 for the experimental group (n = 15), and an average change of -1.375, a standard deviation of 2.93 for the control group (n = 16). A two-sample t-test at 95% confidence interval yielded a p-value of 0.3246.

Conclusion

Although this trial did not demonstrate a statistically significant difference in the average score change, the increased exposure to both surgical and anatomical concepts provides a relevant learning experience for students before their clinical curriculum. In the future, we aim to integrate our simulator into pre-clinical medical education. Furthermore, we plan to evaluate the impact of our simulator on the performance of the two groups during surgical rotations as part of our ongoing research.

## Introduction

The goal of simulation technology is “to facilitate learning through immersion, reflection, feedback, and practice, minus the risks inherent in a similar real-life experience” [1]. There are few opportunities outside of the operating room that allow for creating a realistic learning environment for medical students in the field of surgery. Simulation learning provides hands-on, focused training, with immediate feedback in a controlled and safe setting [2,3]. A randomized trial conducted in 2014 concluded that first- and second-year residents with access to comprehensive and supervised laparoscopic simulator training significantly improved their cognitive, technical, and non-technical skills compared to their colleagues without such training [4]. This study also suggested that exposure to surgical simulation before residency would enhance pre-clinical medical students’ confidence and learning, thus positively affecting their future surgical rotations [4]. Additional studies have found that technical skills learned in the simulation setting are transferable to clinical practice and provide a superior learning environment for students [5,6]. In 2013, the American College of Surgeons, the Association of Program Directors in Surgery, and the Association for Surgical Education (ACS/APDS/ASE) collectively created a resident preparatory curriculum in response to studies suggesting that first-year residents were not adequately prepared for the tasks and responsibilities of residency [2]. They piloted this curriculum in 49 schools in 2013 and made it available to all schools in 2015 [2]. Today, the ACS/APDS/ASE list of critical content is still used to strengthen the skills of fourth-year medical students entering surgical residencies [7]. As a component of the critical content list, operative anatomy is an element that can be learned most realistically through simulation-based learning during medical school.

Synthetic reality allows a unique and reproducible learning experience in an environment that eliminates potential patient risk. Simulation-based learning also allows students to learn without the distraction of multitasking and feeling overwhelmed. A study in 2009 revealed that interactive learning with simulators enhanced retention by 45% compared to audiovisual learning [8,9]. The shift toward experiential medical training offers students a unique opportunity to learn through various styles. However, high-fidelity simulations, which are computerized and complex configurations of realistic structures, are much more expensive to manufacture. As a result, they are difficult for school systems to acquire and integrate into their curricula [2]. Repetitive practice with simulations is essential for effective skill improvement, and the devices must be accessible during learners’ schedules and available at patient-care facilities and institutions [10]. Therefore, despite advancements in this domain of medical technology, hands-on surgical experience is neither accessible nor available to many medical students receiving didactic education.

To combat this, we created a low-cost abdominal surgery simulator for use during instructional years. By utilizing the simulator, we hope to enhance medical students’ procedural knowledge and success by integrating foundational anatomy with surgical pathology. As the subject of our project, we propose that medical students who use the simulator will gain a better understanding of abdominal anatomy and will demonstrate superior academic performance on assessments and performance during clinical rotations.

## Materials and methods

Trial design

To evaluate the effectiveness of an abdominal surgical simulator on medical students' performance in high-yield gastrointestinal (GI) anatomy, we first developed content-based assessments. The assessments were focused on relevant information pertaining to the Comprehensive Osteopathic Medical Licensing Examination (COMLEX) Level 1 and the United States Medical Licensing Examination (USMLE) Step 1 board examinations that Doctor of Osteopathic Medicine (DO) and Doctor of Medicine (MD) medical students, respectively, must pass before entering supervised clinical rotations. This pilot study was a single-center, balanced randomization with 1:1 allocation, parallel-group study conducted on second-year medical students at the Edward Via College of Osteopathic Medicine-Carolinas Campus (VCOM-CC). Before commencing this study, we obtained IRB approval through the VCOM Institutional Review Board (IRB #2023-046), ensuring this study adhered to ethical standards and that participants’ rights were safeguarded throughout the research process.

After trial commencement, the simulation event was rescheduled from March 4, 2024, to April 8, 2024. This change was performed due to scheduling conflicts with the intended study population on the original simulation date. No other changes were made, including the eligibility criteria or trial design.

Participants

This pilot study recruited medical students attending VCOM-CC. The criteria for inclusion were second-year medical students at VCOM-CC in good academic standing. The exclusion criteria included second-year medical students on this research team. All participants were over 18 years of age. To recruit participants, a clinical faculty member sent a recruitment email to the target study population, second-year medical students at VCOM-CC. The recruitment email described the study’s purpose, expected time commitment, and study logistics. Students interested in joining the study entered their name and professional email into the attached interest form. Once the interest form was closed, a study investigator sent an informed consent form to students who responded. The informed consent form was approved by the VCOM IRB and delineated the research, the study’s purpose, and the risks and benefits of participating. Additional verbal consent was obtained on-site for participants engaging in the simulation event.

The intended sample size for the study was 50 second-year VCOM-CC medical students. The study was available to all 160 students in the VCOM second-year class, in which 43 students initially joined the study and 31 continued participation throughout the study. The minimum number of students for the study was eight, allowing for one student per experimental group module and the remaining four students in the control group. No interim analyses were performed during this project. The project was run continuously, and there were no stopping points.

Interventions

Simulator Development

**Figure 1:**
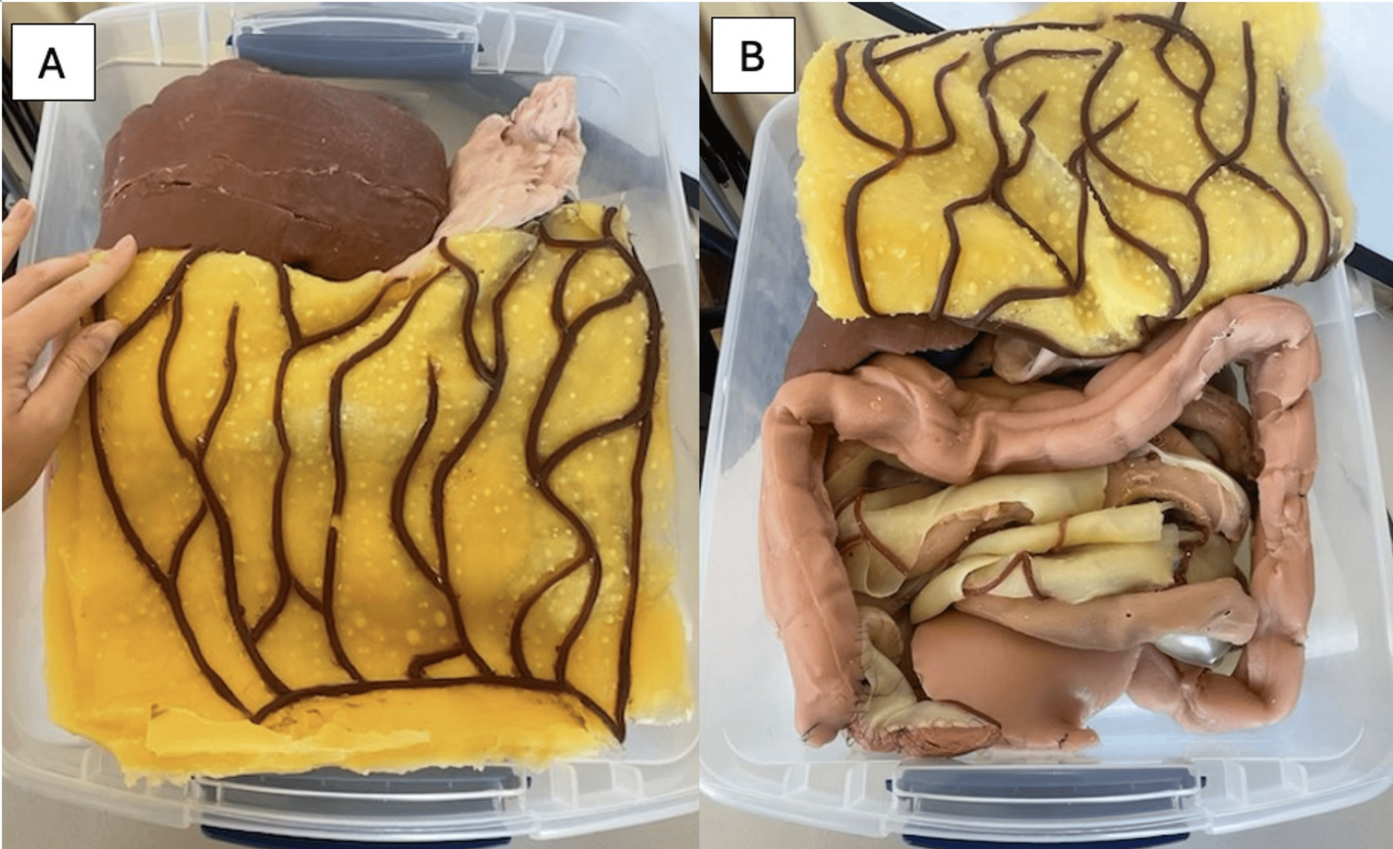
Final simulator without the parietal peritoneum and skin overlay. The simulator with organs and omental layer as positioned for each procedure (A), and the omental layer folded superiorly to showcase the intestines (B). A silicone layer to represent the parietal peritoneum was placed directly over the pictured structures and within the lateral edges of the bin holding the simulator organs. Then, the muscle, subdermal fat, and skin layers, represented by one multitiered silicone surface, were placed over the parietal peritoneal layer and secured along the outside of the simulator bin with Velcro for the procedures.

In this project, we developed a low-cost abdominal surgery simulator using Smooth-On silicone materials and 3D-printed organ models to simulate surgical training during clinical years. The final simulator, shown in Figure 1 without the skin overlay, included all pertinent abdominal cavity anatomy to perform multiple open abdominal surgeries. Structures between the diaphragm and pelvic region were represented to focus on abdominal cavity anatomy. The simulator included structures from the GI system, which began cephalically at the stomach, the female genitourinary system, and the female reproductive system. Detailed structural relationships such as the portal triad and biliary system, the ligaments supporting the uterus, and key abdominal vasculature highlighted the anatomical knowledge necessary to perform abdominal surgeries. Pertinent tissues notable in the abdominal cavity were also exhibited, such as the parietal peritoneum surrounding the entire cavity, the greater omentum, and the mesentery. The total material cost was $2,753.47, excluding the price of the 3D printer owned by the institution. Therefore, the cost-effectiveness of our fully functional abdominal surgical simulator helps to address the increasing need for low-cost real-tissue training devices. To maintain continuity and optimize functionality, the team returned the simulator to its original state before each simulated surgical case. These procedures were guided by a board-certified general surgeon in a simulated operating room.

Assessment Creation

During this study, participants completed a pre-assessment and post-assessment, each consisting of 20 multiple-choice board-relevant GI anatomy questions based on tested content on the COMLEX-USA Level 1 and USMLE Step 1 board examinations. Members of the research team created board-style questions for these assessments by referring to the VCOM-CC GI Anatomy Exam One curriculum objectives provided by the school. The objectives reflect anatomical topics tested on medical school and medical licensing examinations and correlate to structures displayed in the simulator. Researchers used this information to deduce which pertinent objectives about surgical anatomy exhibited weaker class performance. Two questions were generated for each pertinent objective and were randomly distributed between the two assessments. Therefore, the assessments exemplified similar board-relevant information and quantity of topics. The assessments also adequately reflected material examined in medical school curricula and medical licensing examinations without being identical.

Assessments were sent via email as a Google Forms link (Google, Mountain View, CA, US). To submit Google Forms, participants were required to complete every question on the assessment. Participants were not shown their performance upon completion of the assessment. These assessments were held to VCOM Honor Code standards, therefore not tolerating any outside resources or collaboration among students. The first question of the pre-assessment prompted the participants to create a self-generated identification code (SGIC), which was also input into the post-assessment. Therefore, assessments were matched to each participant, providing anonymous longitudinal data collection in the study.

Module Creation

Learning modules dedicated to each procedure were presented to participants before performing the specific simulated procedure. The research team curated these learning modules on Google Slides (Google, Mountain View, CA, US) following a case-presentation format. Each module presented pertinent information, such as the History of Present Illness, a Review of Symptoms, Vitals, Physical Exam, Laboratory Values, Imaging, Differential Diagnosis, and Plan. The Plan section of each module included a short video illustrating the procedure, found on free resources such as YouTube (https://www.youtube.com). Each module also included high-yield clinical and anatomical quick facts surrounding the topic discussed. Board-relevant information was found through medical school curriculum resources such as Boards&Beyond and the First Aid for the USMLE Step 1 2023 book from McGraw Hill, UWorld Step 1 Question Bank from UWorld LLC, and VCOM lecture material. The principal investigator presented each 10-minute module immediately before the specific procedure.

Procedures

**Figure 2:**
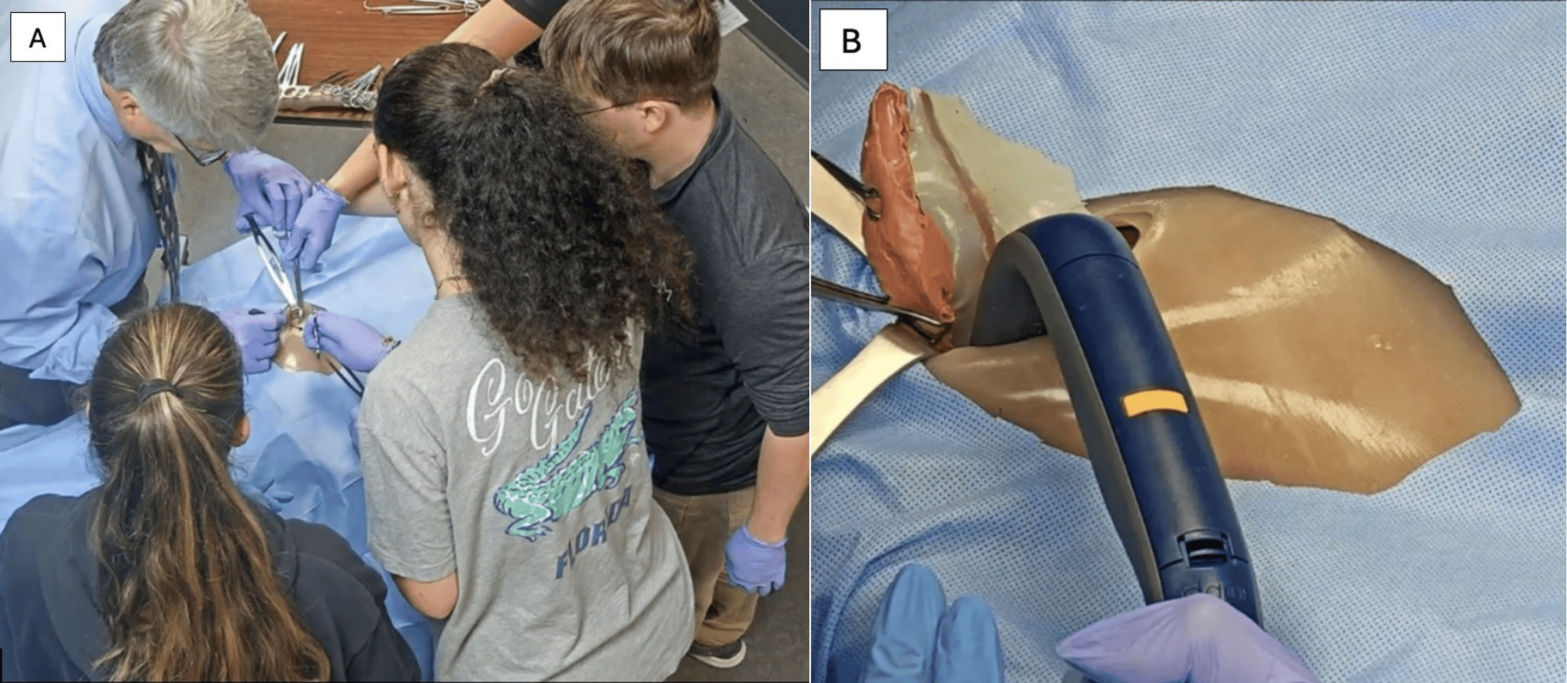
Simulated appendectomy. Performance of the procedure by the principal investigator and assigned participants in the experimental group (A). A still photograph of the live-stream projection of the appendectomy, showcasing the removal of the appendix, mesoappendix, and appendicular artery from the simulator (B).

The trial included an integrative simulation event hosted at the VCOM-CC on April 8, 2024, from 1:00 pm to 5:00 pm. Participants randomly assigned to the experimental group attended this simulation event, with 15 participants in attendance. During the event, the experimental group engaged in an interactive case-based module guided by the principal investigator before each simulated surgical experience. The simulation involved four procedures using the same simulator: appendectomy, cholecystectomy, periumbilical exploratory small bowel obstruction, and hysterectomy with the Pfannenstiel incision. Participants were randomly assigned to one of these procedure groups. Each group performed their assigned procedure under the guidance of the principal investigator, a board-certified general surgeon. The simulation lab was configured to mimic an operating room environment. Participants not actively involved in the procedure synchronously observed the instruction and performance of the procedure via a live-stream video in a separate conference room. Figure 2 shows the appendectomy performed on the simulator and the live-stream shown to participants during the simulation event. Following the simulation event, the experimental group conducted a focus group discussion. This session aimed to gather immediate feedback to minimize recall bias and incorporated subjective questions to gain further insight into the students’ thought processes throughout the simulation event.

Data collection

Data from the pre-assessment and post-assessment were collected using Google Forms links. Scores were calculated by totaling correct responses out of 20 questions. Participants were given one full day to complete each assessment. Data for the pre-assessment were collected from April 7, 2024, at 8:00 am until April 8, 2024, at 12:00 pm. The data for the post-assessment were collected from April 8, 2024, at the conclusion of the simulation event until April 9, 2024, at 5:00 pm. Each participant’s pre-assessment and post-assessment scores were labeled next to their SGIC on a Microsoft Excel sheet (Microsoft Corp., Redmond, WA). Data was only accessible to research team members.

During study development, there were no confounding variables or effect modifiers identified. This study’s design included only two variables: the independent variable, assessment scores, and the dependent variable, exposure to a surgical simulator, precluding the presence of additional variables that could serve as confounders or effect modifiers. Any potential confounding effects on the results were successfully addressed by maintaining consistent difficulty levels between the pre- and post-assessments.

Outcomes

The primary outcome of this study was the average change in scores between the pre- and post-assessment among the two groups. Analysis of collected outcomes began after completion of the post-assessment at 5:00 pm on April 9, 2024. A statistician acquired through VCOM-CC analyzed the data. There were no changes in data collection or analysis of outcomes after commencement, and outcomes remained the same throughout the study.

Randomization

Randomization of the two groups was accomplished using randomization functions in Microsoft Excel. This pilot study used simple randomization based on a 1:1 allocation ratio. Once participants were assigned to the experimental or control group, the experimental group was randomized into four equal blocks. After randomization, group designation was disclosed to participants via email, and participants allocated to the experimental group were invited to attend the simulation event. Study investigators were not blinded to group assignments.

Blinding

This study was not blinded. Participants in the experimental group were notified of their placement to attend the simulation event. Additionally, procedure assignments were revealed to participants during the simulation event. Objective outcomes were used in the study to decrease the risk of bias. Standardization occurred across all four procedural groups to ensure equal exposure to the pre-procedure modules, simulator, and procedures. All participants were presented with the same procedure-centered modules and instructed by the same principal investigator throughout the session. Each procedure was projected live into the main seminar room to ensure participants who were not physically present during the procedure were exposed to the same instruction and direct view of the procedure. Limitations of this standardization include the four procedures being inherently different. Therefore, each procedure group physically utilized the simulator for separate procedures and ultimately had a different experience with the simulator.

Statistical analysis

Analyses began by summarizing the data with counts, sample means, and standard deviations. Given the pre-post nature of the study, we calculated a change score by calculating the difference between post-outcome values and pre-outcome values. We then tested to see if the average change observed per person could be declared statistically significant. We used a two-sample t-test for this hypothesis test using a Type I error rate of 0.05. All statistics and significance tests were calculated/performed using SAS 9.4 (SAS Inc., Cary, NC, US). There were no subgroup analyses or adjusted analyses performed on this data.

## Results

Participant flow

**Figure 3:**
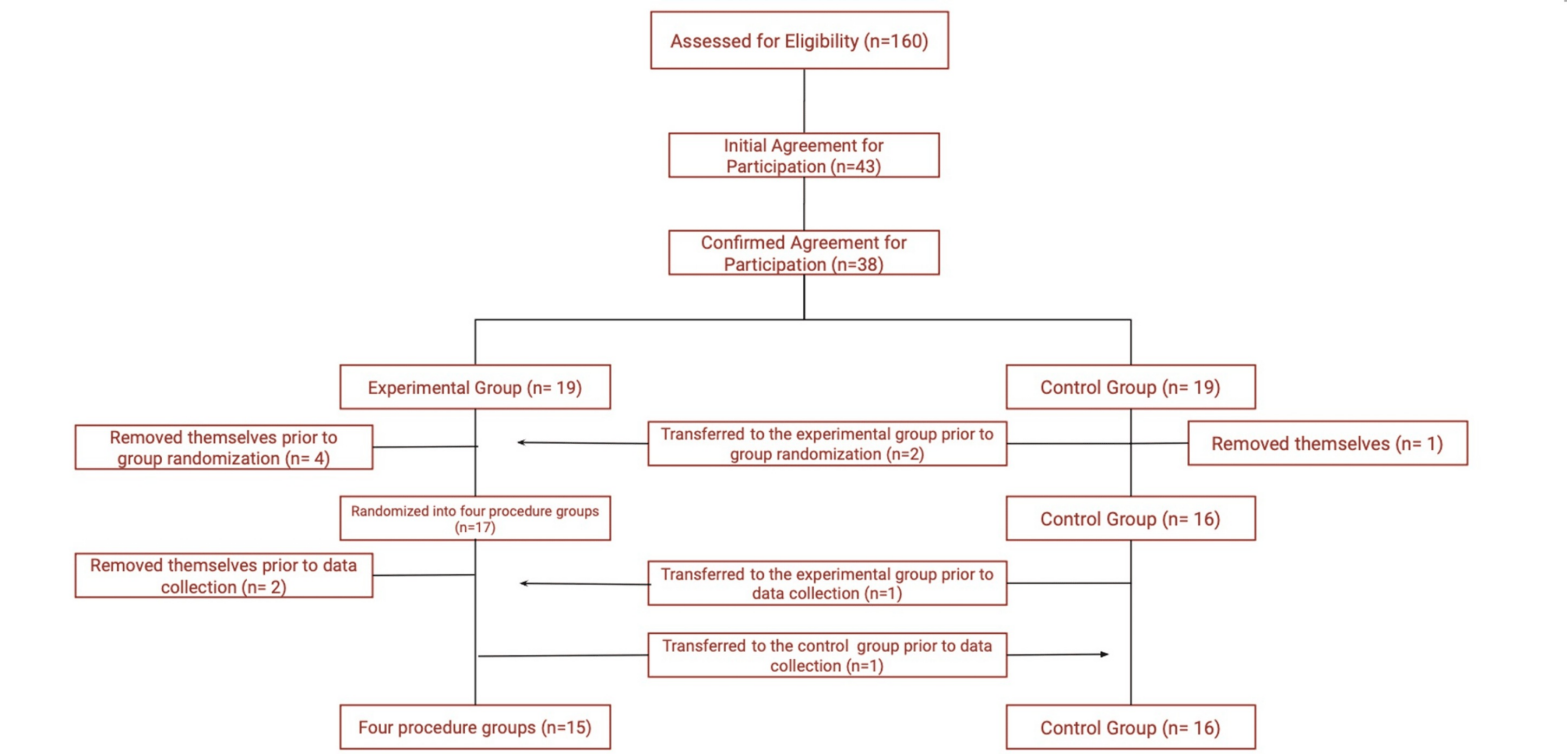
Diagram representing participant flow during randomization. Participant eligibility, participation, and removal from this trial occurred as shown in the figure. A total of six participants removed themselves from the experimental group, and one was transferred to the control group. A total of one participant removed himself/herself from the control group, and three were transferred to the experimental group. Final participation consisted of an experimental group of 15 participants and a control group of 16 participants.

Initially, 43 students filled out the interest form, but five withdrew from the study before the randomization process. This left 38 participants, who were randomly assigned to either the experimental or the control group. Ultimately, only 31 participants remained: 15 in the experimental group and 16 in the control group. Of the 19 assigned to the experimental group, 12 attended the simulation event. Six participants withdrew from this group before data collection-four left before the groups were divided, and two left after. Additionally, one participant who was originally in the experimental group did not attend the simulation but completed both assessments, so they were moved to the control group. In the control group, one participant left before data collection. To ensure the groups were similar in size, three individuals from the control group were randomly selected to join the experimental group. A diagram illustrating these changes is presented in Figure 3. Participants left the study as previously described because of scheduling conflicts.

Baseline data

Baseline demographics in the control and experimental groups were similar, with each group comprising 66.7% women and 33.3% men, and all participants were above 18 years old. Due to all participants following the same medical school curriculum, they had previous exposure to simulation-based learning experiences such as cardiac arrest, labor and delivery, and newborn evaluations. None of the students had exposure to surgical-based simulation through their medical school curriculum. Lastly, all students had relevant anatomy exposure by completing the school’s gross anatomy and didactic anatomy courses during academic pre-clinical training.

Outcomes and estimation

**Figure 4:**
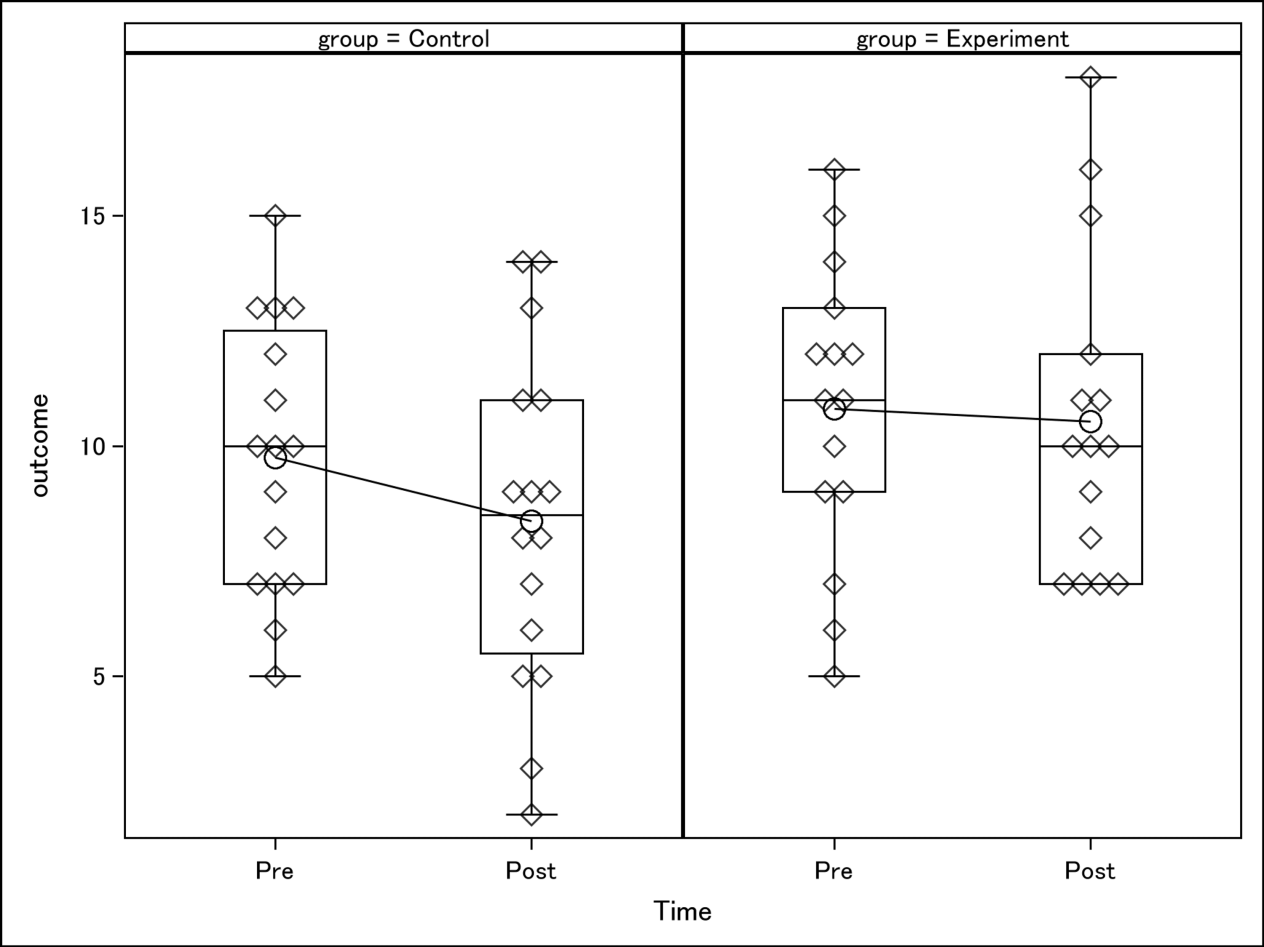
This graph compares the mean test scores between pre-simulation and post-simulation assessments between the control group and the experimental group. A two-sample t-test testing for the difference between groups in average change produced a p-value of 0.3246. Based on the p-value, we conclude there is not sufficient evidence with a two-tailed Type I error rate of 0.05 to conclude the average change differs between groups.

There were 16 individuals in the control group and 15 in the experimental group. For the control group, the average pre-intervention outcome was 9.75, with a standard deviation of 2.93, while the average post-intervention outcome was 8.375, with a standard deviation of 3.65 (Figure 4). Calculating the average change score for the control group revealed an average change of -1.375 and a standard deviation of 2.93. For the experimental group, the average outcome at pre-intervention was 10.80 with a standard deviation of 2.93, while at post-intervention, the average outcome was 10.533, with a standard deviation of 3.46 (Figure 4). Calculating the average change score for the experimental group revealed an average change of -0.267 and a standard deviation of 3.90. A two-sample t-test testing for a difference between groups in average change produced a p-value of 0.3246. Based on the p-value, we conclude there is not sufficient evidence with a two-tailed Type I error rate of 0.05 to conclude the average change differs between groups.

Ancillary analyses

There were no additional analyses performed.

Adverse events

The study involved no harm or unintended effects from participation.

## Discussion

The purpose of this study was to evaluate the effectiveness of an open surgical simulation experience by analyzing the average change in performance on pre- and post-assessments between two groups. Assessments used in this study were built to determine participants' understanding of board-relevant abdominal anatomical concepts. This simulation aimed to replicate a real-life operative room experience for exposure to applicable abdominal structures and relationships as examined throughout undergraduate medical curricula. Through this randomized controlled trial, participants who engaged in the scenario-based training did not demonstrate a difference in average score change between assessments compared to traditional training methods. Both groups showed a decrease in average scores from pre- to post-assessment. However, the experimental group, which participated in the integrated simulation event, experienced a smaller decline in average scores than the control group. These results imply that simulation may effectively improve comprehension and performance on knowledge-based material.

This trial analyzed simulator efficacy for improving academic performance on assessments in contrast to recent studies examining simulator effectiveness on clinical competency, reasoning, and skill. A comprehensive narrative review conducted in 2023 focused on the use of surgical simulation for developing skills and preparing surgery residents for real-life scenarios concluding their usefulness in these competencies [11]. Additional literature published in 2010 surveyed the preferences of internal medicine residents regarding the content, features, and learning experience of simulators [12]. Survey responses also indicated that simulator and instructor-integrated learning subjectively improved clinical techniques, understanding of equipment, and troubleshooting [12]. This survey poses a significant challenge to the objectives of this study, which hypothesized that working with the simulator would improve understanding of knowledge-based components during didactic years. Though this study did not prove statistical significance in the parameter of analysis chosen, it may demonstrate benefit in improving medical student confidence and perceived preparation for post-didactic surgical clinical rotations.

The aforementioned comprehensive narrative review conducted in 2023 primarily involved the use of simulation training during surgical graduate medical education [11]. In contrast, this trial implemented simulation exposure to medical students during their pre-clinical undergraduate medical education. While there is no existing literature indicating that specific demographics will benefit more from simulation training than others, the applicability of the conclusions drawn from this research may not be generalizable to populations with differing knowledge bases and clinical backgrounds.

In June 2024, a comprehensive literature review was performed to determine the impact of simulation-based training (SBT) during medical education [13]. This literature review is comparable in the demographic focus and intended purpose of this study and similarly used parameters of clinical proficiency to analyze the impact of SBT in medical education [13]. Additionally, this review noted that comprehensive post-simulation debriefing sessions, involving targeted feedback on performance, potential mistakes, and areas of improvement, were critical to reinforce learning and correct errors in simulated experiences [13]. Though this trial did feature a post-simulation debrief, the gathering did not provide formal feedback on performance to reinforce learning for the participants. As simulator uses described in this review were similar to this study, implementing a thorough post-simulation discussion would help foster reflective practice and contribute to individual and team development.

The operative simulation experience of this study was organized into small procedure group sizes, providing participants with direct instruction and visualization of anatomical structures. A survey of internal medicine residents on preferences for simulation experiences described an ideal teacher-learner ratio of 1:4 for active engagement and immediate feedback during simulation exercises [12]. This preference aligns with the small group format used in this trial, further supporting the value of the aforementioned operative simulator experience.

Additional pilot studies with a design similar to ours, incorporating both simulation and training modules, demonstrated that students who engaged with both components improved their technical skills and overall surgical performance [14]. The training modules used in this trial provided an overview of surgical techniques and a detailed anatomical and pathophysiological understanding of the diseases represented in the simulator. Furthermore, the training modules in this study were strategically built to address content focused on the study's pre- and post-assessments and high-yield content for board licensing examinations. In contrast, effectiveness in other published trials underscored the importance of surgical proficiency and skill, focusing their training modules on these specific areas [14].

Overall, this study stands out from other studies of similar caliber by concentrating on parameters of effectiveness related to academic performance combined with practical surgical experience. These differences highlight the need for further investigation into the utility of simulated operative experience for a better understanding of operative anatomical concepts in pre-clinical medical education.

Limitations

One significant limitation of this study is its small sample size, which affects its internal and external validity, making it difficult to generalize the findings. The limited sample size was primarily due to the time commitment required for the complete simulation experience. The experimental group had to commit to a four-hour simulation experience, which likely deterred potential participants from joining and remaining in the study. In future studies, we aim to increase the sample size by scheduling the simulation event at a convenient time for the students. The simulation event in this study took place while second-year medical students were preparing for board examinations. Consulting students to determine the best time to hold the simulation could optimize the event timing and minimize scheduling conflicts. Overall, acquiring a larger sample size would greatly increase the power of the study.

Another limitation of this simulation-based research study is the limited applicability of simulated procedures to real-life operating room situations. Simulation-based learning has constraints in transferring to real-world scenarios, as it is a controlled environment, posing a challenge for learners to apply the skills learned in simulation to their workplace. In addition, we recognize the cost of obtaining and adequately compensating faculty for these purposes may limit the study's reproducibility. A final limitation lies in the simulator’s functionality, as its failure during a simulation event could significantly impact the study. Due to the nature of the simulator, it is challenging to control for inherent limitations in this study design. Future studies may incorporate standardization protocols to minimize the risk of simulation malfunction.

Generalizability

The generalizability of this pilot randomized controlled study is limited due to its relatively small sample size. The small sample size also affects the study’s internal and external validity. In addition, the four procedures varied between each group in our experimental group, leading to inevitable differences in instruction. To address this, we live-streamed each procedure, so each participant in the experimental group listened to and watched all four procedures in real time with the same instruction.

## Conclusions

The results did not show a statistically significant average change in test scores between pre- and post-assessments among controls versus experimental groups; however, they demonstrate a subjective benefit in boosting confidence and knowledge through risk-free and hands-on experience. To fully leverage the benefits of simulation training, more research should be dedicated to integrating simulation into the curriculum to maximize its advantages. This research project contributes valid data in support of simulation training for medical education by suggesting long-term benefits despite a lack of measurable immediate improvements in learning outcomes. To better understand how different training methods affect clinical outcomes, we hope to continue exploring the role of surgical simulation in enhancing medical students' practical skills. A future key research goal is to examine how exposure to surgical simulations influences medical students' performance in clinical surgical rotations.

## Appendices

Questionnaires

Pre-assessment

1. A 15-year-old girl is evaluated in the ICU due to nausea and bilious vomiting that started six hours ago. The patient was admitted to the ICU 14 days ago due to a traumatic motor vehicle accident. Since admission, the patient has received continuous IV fluids, analgesics, nutritional support, and wound care. She has been slowly improving until today. Vitals include a temperature of 101.1°F, blood pressure of 140/80 mm Hg, pulse of 110/min, and respirations of 24/min. Physical examination shows mild diaphoresis and mild upper abdominal distention. Blood cultures are negative. CT scan of the abdomen with IV contrast shows significant reduction of the superior mesenteric artery and the aorta, with compression of the intervening portion of the gastrointestinal tract. Impingement of which structure is most likely responsible for the patient’s current condition?

a. First portion of the duodenum

b. Duodenojejunal flexure

c. Pyloric sphincter

d. Fourth portion of the duodenum

e. Third portion of the duodenum

2. A 45-year-old man sustains perforation of the large bowel after a stabbing wound directed posteriorly at the right lower quadrant. An exploratory operation is planned to resect the affected bowel. The surgeon notices that the artery supplying the distal small bowel has been perforated. To prevent excessive hemorrhage in this patient, ligating the terminal branch of which artery is most appropriate?

a. Superior mesenteric

b. Celiac

c. Inferior mesenteric

d. Inferior rectal

e. Left colic

f. Middle colic

3. A 52-year-old woman is admitted to the hospital following a Roux-en-Y gastric bypass 12 hours ago. She alerts her nurse that she is experiencing abdominal pain that started one hour ago. She shares that she noticed bright red blood in her stool. Her procedure was complicated by excessive bleeding that required a transfusion. Vital signs show a blood pressure of 80/50 mm Hg and a pulse of 120/min. A digital rectal examination confirms the presence of bright red blood. An abdominal x-ray shows edematous thickening of the colonic walls of the left colon. This patient’s condition is most likely caused by damage near which gastrointestinal tract structure?

a. Duodenojejunal junction

b. Hepatic flexure

c. Hepatocystic triangle

d. Ligament of Treitz

e. Splenic flexure

4. A 55-year-old man with chronic hepatitis B complains of abdominal pain that has worsened in the past three months. Upon further conversation, he admits to occasional black, tarry stools. Physical examination reveals a frail gentleman with decreased muscle mass, a large central abdomen, and yellow discoloration of the skin and eyes. There is bilateral paravertebral hypertonicity and tenderness from T5-9. Ultrasound of the abdomen shows enlarged lymph nodes in the hepatoduodenal ligament. Which of the following structures is most likely being compressed by these large lymph nodes and causing this patient’s presentation?

a. Common hepatic artery

b. Hepatic ducts

c. Common bile duct

d. Hepatic veins

e. Remnant of the umbilical vein

5. An 18-year-old man arrives at the emergency department complaining of severe abdominal pain. He states this pain started a few days ago but was dull and centered around his umbilicus, before moving to the right lower quadrant when he woke up this morning. Physical examination reveals guarding and rebound tenderness upon palpation of the abdomen. CT scan of the abdomen confirms acute appendicitis. The patient is sent to surgery and the appendix is removed. During the surgery, the appendiceal artery is ligated. The origin artery of this branch also supplies which structure?

a. Hepatic flexure

b. Transverse colon

c. Ascending colon

d. Jejunum

e. Duodenum

f. Descending colon

6. A 47-year-old man arrives at the hospital via ambulance from an apparent gunshot wound to the abdomen. The patient states that he got into an argument with a coworker when he shot him straight on at his left upper abdomen in the seventh intercostal space. Physical examination shows an entry bullet wound on the anterior left upper abdomen and an exit wound on the left upper back. After entering the skin and muscle layers, entering the peritoneal cavity, and piercing through the bottom edge of the liver, which is the next most likely organ the bullet hit?

a. Pancreas

b. Spleen

c. Aorta

d. Stomach

e. Duodenum

f. Splenic flexure

7. A 24-year-old woman arrives at the emergency department following a traumatic penetrating injury to her abdomen. Upon further investigation, the injury penetrates straight into her liver, which starts to bleed profusely into her abdomen. What embryologic structure did this structure arise from?

a. Foregut

b. Midgut

c. Hindgut

d. Urachus

e. Vitelline duct

8. A 78-year-old woman presents to the clinic with right upper quadrant pain. The patient reports this pain came on suddenly and you are worried about colonic ischemia. Which artery allows for the dual supply to the left splenic flexure?

*a. Artery of Drummond off of the SMA* 

b. IMA

c. Celiac trunk

d. Left colic artery

e. Sigmoid artery

9. A 35-year-old woman arrives at the emergency department and reports she has had a fever for the past two days and abdominal pain. She states she feels like she has a mass in her abdomen that’s been progressively getting bigger. On physical examination, there are no abnormalities noted on the patient's stomach. Vitals are notable for tachycardia and a slight fever. A supine CT scan of the abdomen shows a dense pelvic abscess. Where is the most likely site of her pelvic abscess?

a. Right upper quadrant

b. Appendix

c. Pouch of Douglas

d. Along the mesentery

e. Left lower quadrant

10. A 35-year-old woman reports to the office complaining of fullness in her abdomen with some appreciable swelling on physical exam. During the physical examination, you note some tender and swollen lymph nodes in the area of the left and right gastric lymph nodes. Into which structure do these lymph nodes drain first?

a. Thoracic duct

b. Cisterna chyli

c. Celiac lymph nodes

d. Mesocolic lymph nodes

e. Superior mesenteric lymph nodes

11. A 56-year-old patient presents to the office with a long-standing history of alcohol abuse. On physical examination, you note hepatomegaly, jaundice, asterixis, and some swollen veins on his abdomen. Based on the patient’s history, which veins are most likely swollen?

a. Periumbilical and superior epigastric veins

b. Portal vein and hepatic vein

c. Internal iliac vein

d. Internal thoracic vein

e. Femoral vein

12. At what level does the esophagus penetrate the diaphragm?

a. T4

b. T6

c. T8

d. T10

e. T12

13. A patient presents to triage with a gunshot wound to the left abdomen. The patient is in visible distress and states that she feels like she is going to pass out. The patient is brought into the operating room for exploratory abdominal surgery. During surgery, the surgeon discovers the artery coursing along the superior aspect of the pancreas is injured. This artery is most likely the

a. Common hepatic artery

b. Left gastric artery

c. Right gastric artery 

d. Right gastroepiploic artery

*e. Splenic artery* 

14. A 75-year-old man presents to the emergency department with an episode of bloody diarrhea. The patient reports these episodes have been going on for the past two days. A colonoscopy is performed and shows bleeding at the superior lateral segment of the cecum. A branch of which artery was most likely responsible for the bleeding?

a. Celiac artery

b. Inferior mesenteric artery

c. Left colic artery

d. Right internal iliac

*e. Superior mesenteric artery* 

15. A 52-year-old man presents to the office with complaints of a painless mass in his left groin. On examination, several large, hard lymph nodes are palpated in the left inguinal area inferior to the inguinal ligament. An excisional biopsy is performed and malignant cells are identified. The malignant cells found in this region most likely originated from which of the following sites?

a. Dome of the bladder

b. Lateral lobe of the prostate

c. Orifice of the anal canal

d. Upper pole of the testes

e. Upper third of the rectum

16. A patient presents to the emergency department due to severe anal pain. The patient describes noticing streaks of blood on the tissue paper after wiping. He has no abdominal pain, nausea, vomiting, or weight loss. On examination, there are several bulging sacs in the anal canal originating below the dentate line and are tender. The sensory innervation is most likely carried by which of the following?

a. Ilioinguinal nerve

b. Inferior gluteal nerve

c. Pudendal nerve

d. Pelvic splanchnic nerves

e. Inferior hypogastric plexus

17. A patient with a stab wound in the right upper abdominal quadrant is brought into the emergency room. The patient is taken for immediate surgery and approximately 1 L of blood is evacuated from the peritoneal cavity. Brisk, non-pulsatile bleeding is seen emanating from behind the liver. The surgeon decides to use the Pringle maneuver by clamping the portal triad in the hepatoduodenal ligament. However, the patient continues to hemorrhage. Which of the following structures is the most likely source of the patient's bleeding?

a. Common bile duct

b. Cystic artery

c. Hepatic artery

d. Inferior vena cava

e. Portal vein

18. A patient is brought into the emergency room after a motor vehicle accident. Intraperitoneal bleeding is suspected. Which of the following structures is intraperitoneal?

a. Right renal artery

b. Adrenal glands

c. Middle colic artery

d. Ascending colon

e. Distal rectum

19. Which of the following structures would have a serosal layer?

*a. First portion of duodenum* 

b. Second portion of duodenum

c. Adrenal glands

d. Ascending colon

e. Head of pancreas

20. A 78-year-old man comes to the office complaining of a one-day history of left lower quadrant abdominal pain. He has smoked a pack a day for 45 years and eats a diet full of red meat. Temperature is 101°F, blood pressure is 150/110 mm Hg, pulse is 107/min, and respirations are 19/min. CT scan of the abdomen confirms acute diverticulitis. The area of the gastrointestinal tract most associated with this condition receives sympathetic innervation from which structure?

a. Superior mesenteric ganglion

b. Inferior mesenteric ganglion

c. Celiac ganglion

d. Pelvic splanchnic nerve

Post-assessment

1. A 75-year-old man is brought to the emergency department 45 minutes after the sudden onset of tearing chest pain that radiates to his back. He has a 45-pack-year smoking history. His blood pressure is 180/120 mm Hg and his pulse is 120/min. Physical examination and a chest x-ray show no abnormalities. Contrast sagittal CT scans of the chest and abdomen confirm a dissection of the first branch of the abdominal aorta. Occlusion of this vessel will most likely result in compromised blood flow to which of the following organs?

a. Colon

b. Kidney

c. Jejunum

d. Spleen

e. Pancreas

2. A 35-year-old woman is brought to the hospital with acute flank and abdominal pain. A CT scan of the abdomen shows an aneurysm of the proximal portion of the superior mesenteric artery compressing the structures beneath. This aneurysm is most likely to impede venous blood flow from which of the following organs?

a. Liver

b. Right kidney

c. Left kidney

d. Right adrenal gland

e. Left adrenal gland

f. Spleen

3. A 21-year-old man is brought into the emergency department following a motor vehicle accident. Upon arrival, the patient appears stable but complains of mild epigastric abdominal pain. Blood pressure is 110/78 mm Hg, pulse is 102/min, and respirations are 22/min. CT scan of the abdomen reveals a retroperitoneal hematoma. Trauma of which of the anatomical structures is most likely responsible for this hematoma?

a. Jejunum

b. Liver

c. Pancreas

d. Spleen

e. Transverse colon

4. A 35-year-old woman presents for an elective cholecystectomy for pain after meals and gallstones. She denies any current symptoms, and vital signs are within normal range. On physical examination, the abdomen is soft and non-tender. She proceeds to surgery, and during the cholecystectomy, she becomes hypotensive and tachycardic following a vascular injury. The gallbladder wall is pale, and bleeding is noted in the localized area around the gallbladder. Which of the following arteries is the most appropriate to clamp to temporarily stop the bleeding and repair the injury site?

a. Celiac trunk

b. Gastroduodenal artery

c. Right gastric artery

d. Right gastroepiploic artery

e. Common hepatic artery

5. A 23-year-old woman arrives at the emergency department complaining of severe abdominal pain that started this morning. The pain was originally centered around her umbilicus when it started a few days ago but recently moved to the right lower quadrant. Physical examination reveals guarding and rebound tenderness upon palpation of the abdomen. CT scan of the abdomen confirms acute appendicitis. Which is true regarding the structure the appendix extends from?

a. It is innervated by the inferior mesenteric ganglion

b. It receives blood supply from the right colic artery

c. It is intraperitoneal

d. It is retroperitoneal

e. It receives blood supply from the middle colic artery

6. A 65-year-old man arrives at the emergency department due to two episodes of bloody vomit that started this morning. Past medical history is notable for cirrhosis secondary to alcoholism, hypertension, and tobacco use disorder. Associated symptoms include stools that are dark and sticky for two days. Physical examination is notable for a distended abdomen and yellowing of the eyes. Vitals show a temperature of 98°F, blood pressure of 83/62 mm Hg, pulse of 96/min, and a respiratory rate of 16/min. Labs reveal iron deficiency anemia and metabolic acidosis from blood loss and volume loss, respectively. He is taken for endoscopic evaluation, which shows engorgement of vessels within the esophagus. Which portal vein is most likely associated with this condition?

a. Splenic vein

b. Azygous vein

c. Paraumbilical vein

d. Left gastric vein

e. Right gastric vein

7. A 35-year-old woman presents to the office with epigastric pain that radiates to the right shoulder after consuming certain meals. She states these symptoms start 30 minutes after eating and are worst when she treats herself to her favorite fast-food meal. On physical examination, the abdomen is tender in the right upper quadrant with pain on palpation. Ultrasound of the gallbladder does not show any stones. The physician determines the patient has acalculous gallbladder disease. There is obstruction of bile flow at the point of entry into the gastrointestinal tract from necrotic tissue lodged in the sphincter of Oddi. Which of the following locations does the common bile duct normally release bile into the gastrointestinal tract?

a. Descending portion of the duodenum

b. First portion of the jejunum

c. Pyloric stenosis of the stomach

d. Transverse portion of the duodenum

e. Ascending portion of the duodenum

8. A 56-year-old woman presents to the emergency department complaining of nausea, vomiting, and diarrhea that started roughly 16 hours ago. The patient reports she first noticed the pain lower in her stomach but now describes diffuse abdominal pain. On physical examination, abdominal tenderness is noted diffusely, but no rebound tenderness or guarding is noted. Rovsing and McBurney’s points are both negative. An ultrasound of the right upper quadrant is also negative. Complete blood count shows an increased WBC count. Her vitals show a temperature of 101°F and blood pressure of 133/72 mm Hg. What is the most likely diagnosis?

a. Appendicitis

b. Cholelithiasis resulting in blockage of the pancreatic duct

c. Cholecystitis

d. Spread of infection through the paracolic gutters

e. Ulcerative colitis

9. A 45-year-old obese patient undergoes gastric sleeve surgery. While in the surgery, the surgeon accidentally knicks the short gastric arteries feeding the fundus of the stomach. What artery should he clamp that feeds off the celiac trunk to gain control of the bleeding in this patient?

a. Common hepatic artery

b. Left gastric artery

c. Right gastroepiploic artery

d. Splenic artery

e. Gastroduodenal artery

10. A 56-year-old man reports to the office complaining of a burning sensation in his abdomen after eating spicy foods and when lying down shortly after meals. Physical examination shows epigastric tenderness. On osteopathic examination, it is noted that the patient has somatic dysfunctions from T5-9. What embryologic derivative does the causative organ arise from?

a. Foregut

b. Midgut

c. Hindgut

d. Urachus

e. Vitelline duct

11. A 47-year-old woman arrives at the office complaining of losing weight and feeling fatigued for several months. Further examination shows general epigastric abdominal tenderness upon palpation. A biopsy is taken during an esophagogastroduodenoscopy and shows Gram-negative curved rods inhabiting the portion of her stomach immediately proximal to the opening to the duodenum. Triple therapy is started, and the patient improves. What area of the stomach is this organism most likely to inhabit?

a. Fundus

b. Lesser curvature

c. Greater curvature

d. Pyloric antrum

e. Cardioesophageal junction

12. A 17-year-old patient presents to the clinic complaining of sharp paraumbilical pain that started acutely a few hours ago. Past medical history is noted for a previous acute appendectomy. Surgical exploration begins, and a bowel obstruction is visualized. The obstruction is noted to be 4 in proximal to the ileocecal valve. Which embryological structure is the involved organ derived from?

a. Foregut

b. Midgut

c. Hindgut

d. Left lower quadrant

e. Right lower quadrant

13. A young girl presents to the emergency room with a four-hour history of periumbilical pain and nausea. She initially attributes the symptoms to eating “bad food” at a birthday she was at; however, she states the pain has progressively worsened. On the physical examination, there is right lower quadrant tenderness and guarding. Surgical intervention is recommended. During surgery, which of the following landmarks is most helpful in identifying the symptomatic organ?

a. Greater omentum

b. Lesser omentum

c. Haustra of the colon

*d. Teniae coli* 

e. Psoas major muscle

14. A patient is brought to the emergency room after a motor vehicle accident in stable condition but complains of abdominal pain. The abdominal pain is localized to the epigastrium. CT scan reveals a retroperitoneal hematoma. Trauma of which of the following anatomic structures is likely responsible for these findings?

a. Stomach

b. Descending portion of duodenum

c. Jejunum

d. Superior portion of duodenum

e. Pyloric sphincter

f. Transverse colon

15. A 64-year-old man with a history of alcohol use disorder presents to the emergency department with bright red blood per rectum. The patient denies any pain and states that he first noticed the blood in his stool this morning. On physical examination, the abdomen is non-tender; however, dilated tortuous vessels are noted around the umbilicus. The patient refuses a digital rectal exam. The most likely cause of his current gastrointestinal symptoms involves which of the following vessels?

a. Left gastric vein and azygos vein

b. Paraumbilical vein and superficial epigastric vein

c. Paraumbilical vein and small epigastric veins

d. Superior rectal vein and inferior mesenteric vein

e. Superior rectal vein and inferior rectal vein

16. A 65-year-old man with a history of hypertension and tobacco smoking reports to the hospital with mild back pain. Abdominal examination reveals a bruit but no pulsatile mass. An ultrasound is notable for a large infrarenal abdominal aortic aneurysm. During the repair procedure, the inferior mesenteric artery is ligated, the diseased portion of the aorta is dissected, and a graft is placed from below the renal arteries to the bifurcation of the aorta. Collateral circulation from which of the following arteries is responsible for preventing ischemia of the descending colon?

a. Celiac trunk

b. External iliac artery

c. Internal iliac artery

*d. Superior mesenteric artery* 

e. Renal artery

17. A 21-year-old woman presented to the emergency room with abdominal pain, nausea, and vomiting. The patient described vague periumbilical pain in the morning. Over the next several hours, the pain became more severe, sharper, and localized to the right lower quadrant. On examination, maximal tenderness is found at McBurney’s point and there is a positive Rovsing sign. The change in this patient’s pain characteristics is most likely explained by which of the following?

a. Involvement of the obturator internus muscle

b. Inflammation of the psoas major muscle

c. Irritation of the parietal peritoneum

d. Stimulation of the cecal nerve endings

e. Retrocecal orientation of the appendix

18. A patient presents to the clinic with a two-day history of crampy abdominal pain and vomiting. The patient has a history of hypertension, coronary artery disease, and an episode of acute calculous cholecystitis that was managed non-operatively several months ago. A physical examination shows a distended, tympanic abdomen with high-pitched bowel sounds and right lower quadrant pain. Abdominal x-ray shows air in the gallbladder and biliary tree. The patient's gallstone has most likely lodged in which site?

a. Common bile duct

b. Cystic duct

c. Duodenum

d. Ileum

e. Jejunum

19. A 68-year-old man comes to the emergency department due to several episodes of bright red blood per rectum. Past medical history is notable for a recent diagnosis of diverticula from a recent colonoscopy. Laboratory studies show anemia with a normal coagulation profile. An abdominal angiogram shows active bleeding from the sigmoid colon. Catheter embolization is planned, with entry from the femoral artery. During the procedure, in which order is the catheter most likely to proceed through arteries to the diseased portion of the bowel?

a. External iliac, internal iliac, internal pudendal

b. External iliac, internal iliac, middle rectal

c. External iliac, common iliac, abdominal aorta, inferior mesenteric

d. External iliac, common iliac, abdominal aorta, superior mesenteric

e. External iliac, common iliac, abdominal aorta, celiac

20. A 65-year-old man is evaluated due to several months of abdominal pain and vomiting. The pain starts two to three hours after meals and is associated with bilious vomiting. A CT scan of the abdomen shows an irregular mass in the third part of the duodenum infiltrating beyond the gut wall. If the mass continues to enlarge, which structure will most likely be compromised?

a. Common bile duct

b. Gastroduodenal artery

c. Portal vein

d. Superior mesenteric artery

e. Ureter
